# Combination of Laser Material Deposition and Laser Surface Processes for the Holistic Manufacture of Inconel 718 Components

**DOI:** 10.3390/ma11071247

**Published:** 2018-07-20

**Authors:** Jon Iñaki Arrizubieta, Magdalena Cortina, Jose Exequiel Ruiz, Aitzol Lamikiz

**Affiliations:** Department of Mechanical Engineering, University of the Basque Country, Plaza Torres Quevedo 1, Bilbao 48013, Spain; magdalena.cortina@ehu.eus (M.C.); joseexequiel.ruiz@ehu.eus (J.E.R.); aitzol.lamikiz@ehu.eus (A.L.)

**Keywords:** laser, additive manufacturing, laser beam machining, laser polishing, waviness, roughness, Inconel 718

## Abstract

The present work proposes a novel manufacturing technique based on the combination of Laser Metal Deposition, Laser Beam Machining, and laser polishing processes for the complete manufacturing of complex parts. Therefore, the complete process is based on the application of a laser heat source both for the building of the preform shape of the part by additive manufacturing and for the finishing operations. Their combination enables the manufacture of near-net-shape parts and afterwards removes the excess material via laser machining, which has proved to be capable of eliminating the waviness resulting from the additive process. Besides, surface quality is improved via laser polishing so that the roughness of the final part is reduced. Therefore, conventional machining operations are eliminated, which results in a much cleaner process. To validate the capability of this new approach, the dimensional accuracy and surface quality as well as the microstructure of the resulting parts are evaluated. The process has been validated on an Inconel 718 test part, where a previously additively built-up part has been finished by means of laser machining and laser polishing.

## 1. Introduction

Laser Material Processing is an alternative to many traditional manufacturing processes, such as arc welding, electrochemical machining, hand polishing, electron beam welding, etc. Laser Material Processing’s main characteristic is the use of a high-power laser as a heat source, which results in a very high concentration of the energy density that reduces the Heat Affected Zone (HAZ) and thermally induced distortions [1].

One of the laser-based processes that is experiencing a continuous growth is the Laser Metal Deposition (LMD). This additive manufacturing (AM) technique consists on generating a melt pool on the surface of the substrate, while wire or powder shaped filler material is added simultaneously [2]. Besides, LMD enables to obtain near-net-shape parts, which reduces the amount of wasted material [3,4]. Regarding environmental impact considerations, if material reductions as high as 50% with respect to the initial part are required during the manufacturing process, AM becomes environmentally friendlier compared with machining and forging [5]. In the same way, the aeronautical industry uses the buy-to-fly ratio as an efficiency factor, since it relates the weight of the part that really flights with the weight of the initial part stock. Laser Material Deposition can reduce the buy-to-fly ratio below 1.5:1, comparable to laser welding processes [6]. However, LMD manufactured parts do not meet the final surface roughness and dimensional requirements, and a finishing operation is always required [7]. Usually, conventional machining is applied for the finishing of the parts.

Another laser-based process that has found a niche in the market is the Laser Beam Machining (LBM), where the laser beam is directly applied for melting and vaporizing unwanted material from the substrate surface [8]. As the LBM is a laser-based process, no cutting tools are required, and materials can be machined regardless their hardness [9]. In addition, LBM process applies a laser beam (usually smaller than 75 μm beam diameter) directly for removing surface material. Hence, this process is especially suitable for the machining of small details on hard materials [10]. Moreover, high aspect-ratio grooves and holes can also be achieved [11] and almost no HAZ is generated when nano or femto pulse-duration lasers are used [12]. Nevertheless, as Dubey et al. stated, LBM process is not fully developed, and it is still waiting to its industrial use [8].

LBM does not always provide the desired surface quality and a finishing operation is therefore required. To this end, highly skilled operators using abrasive tools have traditionally performed finishing operations manually. For instance, Peng et al., proposed the Abrasive Flow Machining for removing the falling effect and the powder adhesion generated during AM [13].

An alternative to reduce the surface roughness of previously manufactured parts, which has caught the interest of many researchers, is the laser polishing (LP) [14,15,16]. In LP, peaks of the surface roughness are melted, and the material is redistributed in the valleys due to the surface tension and the gravity [17]. Therefore, when laser-polishing material is not removed, nor the final shape of the part is modified, but material is relocated while melted. To improve the understanding of the effect of LP on additively manufactured parts, Marimuthu et al., studied the influence of the melt pool dynamics on the resulting surface topology and roughness [18].

Other authors have studied experimentally the improvement of the surface quality when AM and LP are combined. For example, Zhihao et al., studied the surface roughness reduction of additively built-up parts using LP [19]. They concluded that LP improves the surface roughness of Inconel 718 Selective Laser Melting manufactured parts. On the other hand, Ma et al., also studied the improvement of the surface roughness of additively manufactured Ti alloys [17]. Nevertheless, the reference surface on which authors applied LP was a W-EDM cut surface and not the wavy surface characteristic of AM.

Up to now, the roughness and excess material resulting from the AM process is eliminated mechanically via milling or other abrasive processes, such as grinding. In this direction, the current trend of modern industry is to combine additive and subtractive technologies within the same machine [20]. However, laser-based processes are not always easily combined with other manufacturing techniques. For instance, the combination of LMD with milling or turning may result problematic, especially when cutting fluids are used. The problems arisen can be classified in two groups. On the one hand, the handling and filtering of the moisture generated when the powder particles and the cutting fluid are mixed results problematic. On the other hand, pore phenomena do appear if the surface is not properly cleaned before the LMD process [21].

Consequently, if LMD, LBM and LP processes are combined, the machining operation could be eliminated from the production chain, which leads to a much cleaner and environmentally friendlier manufacture. Moreover, the use of coolants, tooling, etc. is eliminated, which simplifies the management of the generated residues during the manufacturing process. 

To demonstrate the validity of this statement, a novel manufacturing procedure, fully based on laser, which combines LMD, LBM and LP technologies is developed, where Laser Beam Machining is employed for removing the overstock and waviness generated by Laser Material Deposition. Finally, LP is used for reducing the roughness resulting from the LBM process. Topographies of the attained surfaces are obtained for each operation and roughness values as well as the microstructure are analyzed to evaluate the surface quality.

## 2. Materials and Methods

The proposed process involves very different laser operations. On the one hand, LMD is usually carried out with Continuous Wave lasers, while LBM and LP are usually performed with pulsed lasers. On the other hand, laser beam diameters for LMD are usually between 100 μm and 1 mm, while LBM and LP processes used to be carried out with much smaller laser beams (usually below 75 μm). Therefore, two different machines have been used to perform the proposed procedure. Firstly, the Kondia Aktinos 500 laser center (Kondia, Elgoibar, Spain) coupled with a 1 kW Rofin FL010 fiber laser (ROFIN-SINAR Laser GmbH, Bergkirchen, Germany) has been employed for the LMD tests. The LMD head includes a 200 mm focal length lens that concentrates the laser beam in a 0.75 mm diameter spot, values provided by the laser supplier. Powder material is supplied using a Sulzer Metco Twin 10 C powder feeder (Oerlikon Metco, Pfäffikon, Switzerland) and focused by an in house designed coaxial nozzle, denominated as EHUCoax-2015 (UPV/EHU, Bilbao, Spain) [22]. Argon has been used as protective and carrier gas. Then, a Trumpf TruMark Station 5000 (Trumpf, Ditzingen, Germany) is used for the LBM and LP operations [23]. This marking station has a fiber laser with a Q-switch pulse technology that concentrates a 50 W laser power in 7–500 ns duration pulses. A 2D galvanometric scanner (Trumpf, Ditzingen, Germany) controls the laser beam position and focuses it at a 212 mm focal distance and a 45 μm diameter; these values are supplied by Trumpf (Ditzingen, Germany).

The material used for the tests is Inconel 718 superalloy, which is supplied by Oerlikon Metco (Pfäffikon, Switzerland) under the name MetcoClad 718. The chemical composition of the powder material is shown in Table 1 and, as it can be observed, it is similar to that of Inconel 718. Powder is supplied with a particle size between 44 and 90 microns in diameter and the spherical shape of the particles is ensured as they are manufactured via Argon-gas atomization.

Before manufacturing a final test part, three types of tests are performed:

(1) First, a preliminary test (Test Part 1) for evaluating the capability of LBM for machining LMD manufactured Inconel 718 parts is performed. For this purpose, a 3 mm thickness layer is deposited by means of LMD. Afterwards, the surface of the deposited material is grinded to ensure a flat reference surface. On this surface, different LBM parameters are tested, and, in each case, the reached depth and the resulting surface quality are evaluated. Based on the obtained results, the maximum effective depth at which the laser could remove material is defined.
(2) Secondly, following the same procedure and based on the results obtained in Test 1, the capability of LP for improving the roughness resulting from LBM is evaluated. Based on these results, the optimum LP parameters are defined. Besides, the recast layer generated by LP is measured.
(3) Finally, the capability of LBM for eliminating the surface waviness resulting from LMD is evaluated. In this case, no intermediate grinding operation is performed.

**Figure 1:**
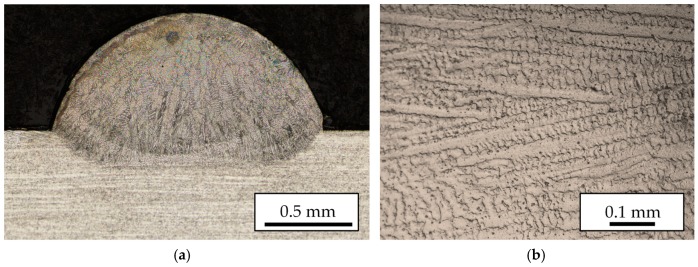
(**a**) Cross section of a single clad; (**b**) Detail of the microstructure.

Process parameters for LMD of MetcoClad 718 were obtained in a previous work [21] and they are detailed in Table 2. In Figure 1 a cross section of a single clad is shown, where the dimensions and dilution can be observed. The sample is etched using Kalling’s 2 reagent to reveal the microstructure originated during the cooling stage. Generated clads have 2 mm width and a constant 0.8 mm height is obtained with each layer.

**Figure 2:**
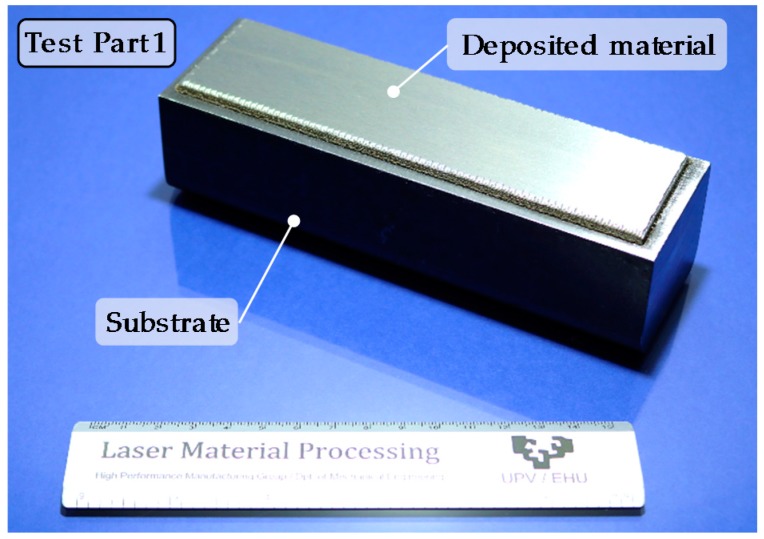
Test part 1 after the LMD and grinding processes.

For the first test, material is deposited over an AISI 1045 substrate. This substrate has no influence on the subsequent LBM operations since they are performed only in the LMD zone. Nevertheless, for the final tests, Inconel 718 substrate is used. Figure 2 shows the substrate with the deposited area after the grinding operation. 

**Figure 3:**
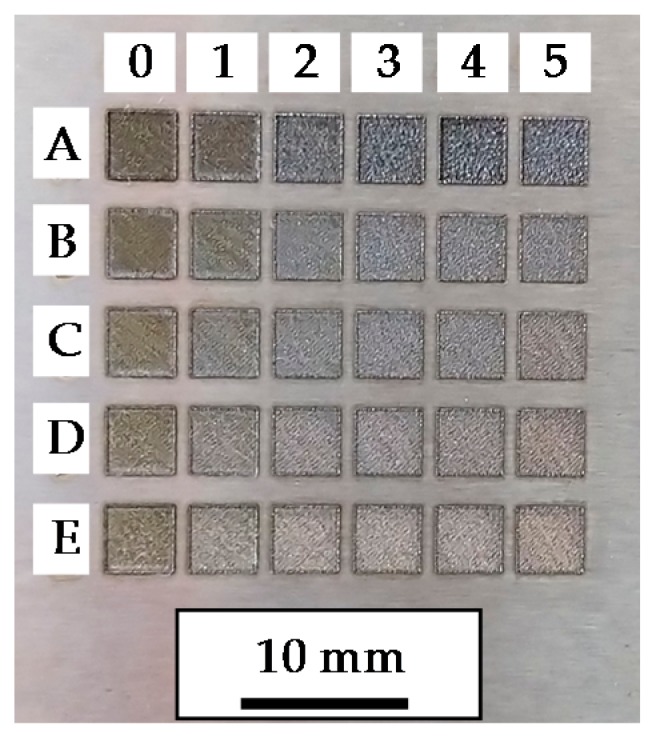
Parameter tests for obtaining the best LBM conditions (Test Part 1).

To determine the best conditions for LBM, a parameter scanning is performed over the grinded flat surface in Test Part 1. Obtained results are shown in Figure 3, whereas the employed parameters in these tests are shown in Table A1 (see Appendix A). 

**Figure 4:**
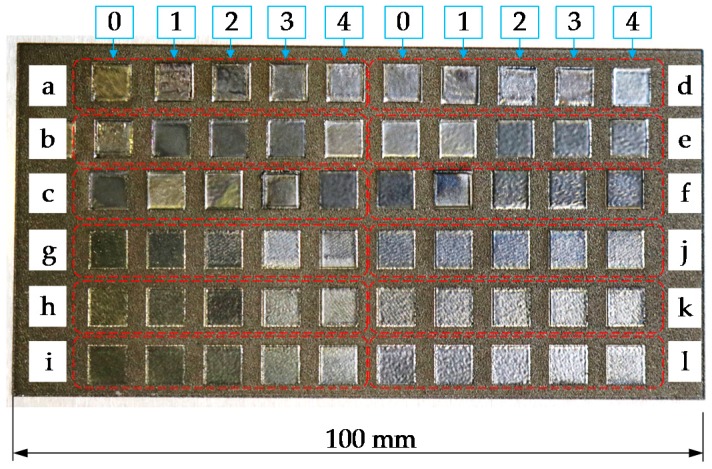
Parameter tests for obtaining the best LP conditions (Test Part 2).

Likewise, with a view to determining the best LP conditions, a parameter scanning has been performed over the LBM surface resulted from applying the optimum process conditions determined previously, see Figure 4. Test codes for the LP tests are named with lower case letters to avoid misunderstandings with the LBM test naming. The parameters of these tests are showed in Table A2 (see Appendix B). 

**Figure 5:**
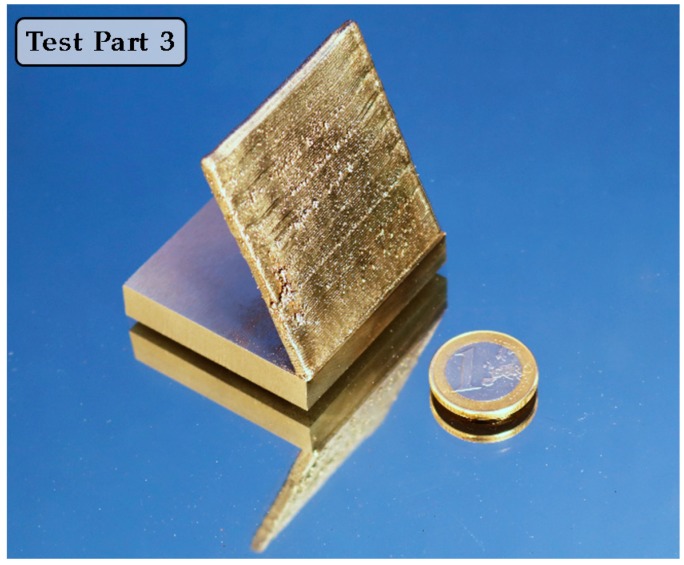
Part manufactured via LMD for Test Part 3.

Once Tests 1 and 2 are carried out and the optimum parameters for LBM and LP are defined for an LMD manufactured Inconel 718 part, Test 3 is performed. Test Part 3, which is also used for the manufacture of the Final Test Part, is manufactured layer-by-layer via LMD and the result is a 50 mm high wall with a 4 mm thickness and 60° inclination, see Figure 5. Please note that in this test, no grinding operation is executed and surface waviness resulting from the LMD process is eliminated exclusively via LBM.

## 3. Results and Discussion

### 3.1. Material Removal via LBM

The aim of the LBM operation is to remove as much material as possible from the substrate. Therefore, to determine the optimum parameters for the LBM operation, the depth reached in each case is measured based on the average surface profiles obtained by means of a Leica DCM 3D confocal microscope. The depth reached in each case after a single repetition is detailed in Table 3, whereas the process conditions employed in each test are detailed in Table A1 (see Appendix A).

Process parameters corresponding to the test A4 are considered the best in terms of penetration and low recast layer; therefore, these parameters are employed for the following LBM operations, see Table 4 and Table 5.

**Figure 6:**
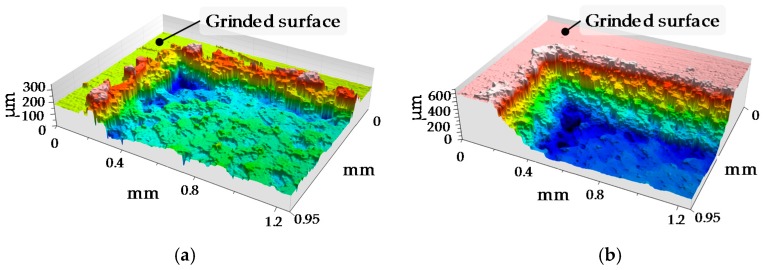
Topographies of the mark after (**a**) One repetition; (**b**) 10 repetitions.

Once the process parameters are determined, LBM is performed on the surface of the Test Part 1, with the laser beam focused on its surface and without changing the focal position between the consecutive repetitions. After every 10 repetitions, the mark generated on the surface of the substrate is analyzed by means of a Leica DCM 3D confocal microscope. In Figure 6, the topographies of two different marks are shown.

**Figure 7:**
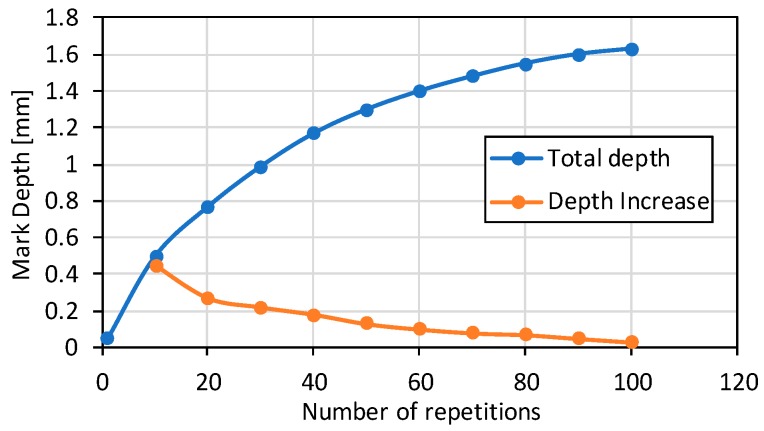
Depth increase as the number of repetitions is increased.

As the number of repetitions is increased, the depth increment is lower, and after 100 repetitions, it is noticed that the laser is not capable of removing material anymore. Therefore, the LBM process is concluded to be capable of removing material until a 1.6 mm maximum distance from the focal plane position (fpp), see Figure 7. It must be highlighted that the laser beam is focused on the original grinded surface of the substrate and its position remains unchanged as the number of repetitions is increased.

Besides, the time required for processing a 1 cm^2^ area until a 0.08 mm depth is of 507.31 s, which results in a 8.3 × 10^−^^3^ g·min^−1^ material removal rate in the LBM process.

### 3.2. Roughness Reduction via LP

In Test 2, the roughness resulting from Test 1 is reduced via LP. With the aim of determining the optimum LP parameters, the resulting Ra roughness is measured in every polished square shown in Figure 4. Roughness measurements are performed according to the standard ISO 4287 and using a Leica DCM 3D confocal microscope. The resulting Ra values in microns are detailed in Table 6, whereas the process conditions employed in each test are shown in Table A2 (see Appendix B).

After the results analysis, it is concluded that for the same process parameters (laser power, frequency, hatching, defocus, etc.) an increase of the laser scan velocity results directly in higher surface roughness. Besides, it is also noticed that the surface roughness improves as the laser frequency is increased, but 200 kHz becomes a limit value, after which Ra value increases.

Regarding the number of hatches used for polishing, the resulting roughness value is improved as the number of repetitions is increased and a lower Ra value is obtained in all cases with 10 repetitions rather than with 5. However, when the number of repetitions is further increased, until 20, there is no considerable roughness reduction, whereas the required time for the process is doubled. Hence, it is decided that 10 repetitions is the optimum parameter tested.

Process parameters corresponding to the test b2 provided the lowest roughness value, and therefore, these parameters are employed for the following LP operations, see Table 7 and Table 8. Notice that due to the 4 mm defocusing, the laser spot becomes approximately 120 microns in diameter at the working plane.

**Figure 8:**
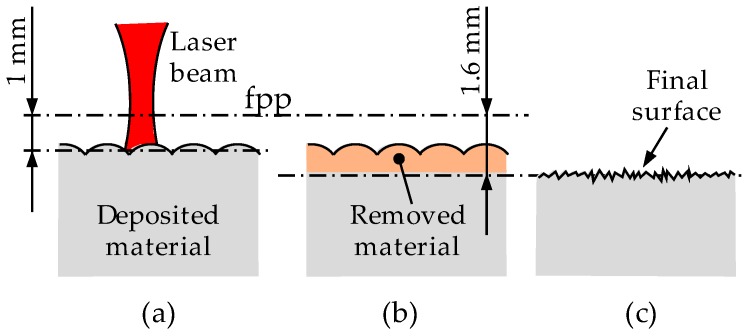
Followed strategy in LBM for obtaining a flat surface from the waved LMD surface. (**a**) LMD manufactured part; (**b**) Material removal via LBM; (**c**) Resulting flat surface after LBM.

The idea of combining LMD and LBM processes arises as a methodology aiming to remove the surface waviness that LMD generates, and thus, obtain a flat surface. To that end, the laser is defocused 1 mm above the desired final surface. Therefore, the laser eliminates all material until a distance of 1.6 mm from the focal plane position, see Figure 8, and the process does only affect material located in this concrete region. However, as the resulting surface quality from the LBM process has a high roughness value, a polishing stage is afterwards performed.

**Figure 9:**
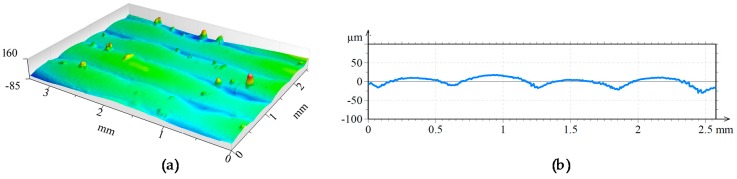
(**a**) Topography and (**b**) surface profile LMD.

**Figure 10:**
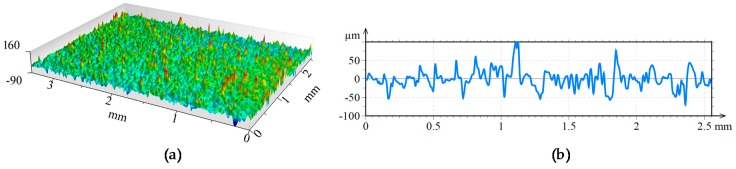
(**a**) Topography and (**b**) surface profile LMD + LBM.

**Figure 11:**
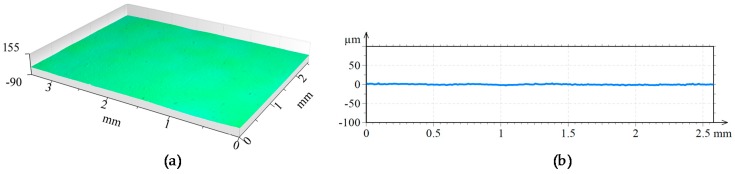
(**a**) Topography and (**b**) surface profile LMD + LBM + LP.

A 3D view of the surfaces attained after the different laser-based processes are shown in Figure 9, Figure 10 and Figure 11. In the three figures, the same height axis scale is used to make results visually comparable. In the case of the LMD surface, roughness is measured perpendicularly to the LMD direction, because LMD is a directional process and so is the resulting surface pattern. On the contrary, in LBM and LP the hatching direction is changed in every repetition to avoid any directional pattern on the surface, and therefore, roughness is independent from the measured direction.

In a second step, to compare numeric roughness values, the roughness of each surface is obtained. For this purpose, the arithmetic mean deviation of the surface roughness (Ra) of five different profiles is measured in each surface and the average value is calculated. Measurements are performed according to the standard ISO 4287. As it is shown in Table 9, the Ra value is higher after the LBM process, than that after LMD. However, LBM provides a waviness-free surface, but the roughness needs to be reduced with the subsequent polishing stage.

### 3.3. Influence of the LP on Material Microstructure

**Figure 12:**
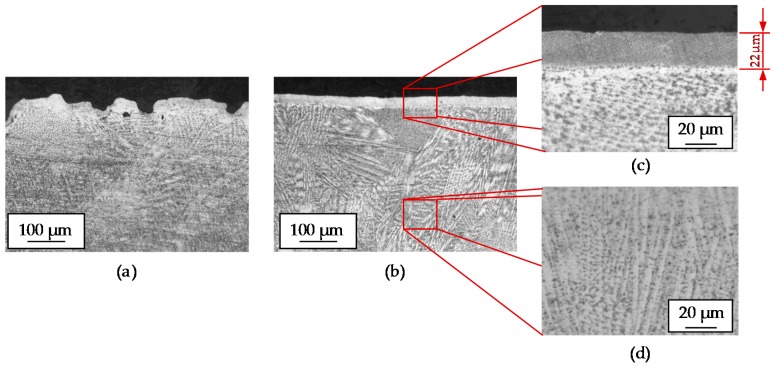
(**a**) Cross section of the LBM manufactured surface; (**b**) Cross section of the LBM + LP manufactured surface; (**c**) Detail of the recast layer generated on the surface due to polishing; (**d**) Internal microstructure developed as a result of the LMD process.

LP is proved to be capable of modifying the surface roughness. However, it also affects the microstructure of the material and generates a recast layer that may modify the mechanical properties of the final part. To evaluate the influence of the LP on the microstructure, both LBM and LBM + LP surfaces have been cross-sectioned, polished, and etched using Kalling’s 2 reagent. Notice that the polished surface shown in Figure 12b is the same LBM surface shown in Figure 12a that has been later subjected to LP. The thickness of the recast layer due to the polishing is of 22 µm, which is a circumstance to be considered depending on the final application of the part.

### 3.4. Final Test Part

**Figure 13:**
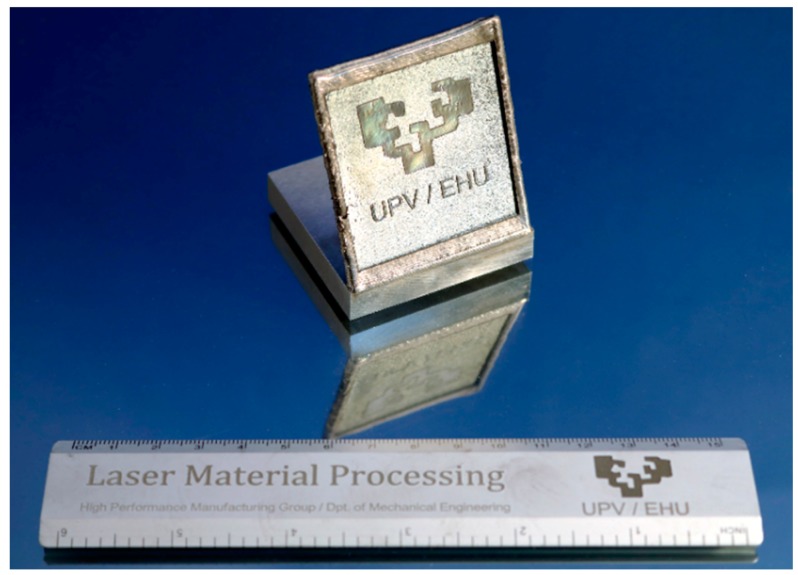
Final shape of the manufactured final test part.

To demonstrate the potential of combining LMD, LBM and LP processes, a final test part is manufactured, Figure 13. First, starting from an Inconel 718 substrate, an oblique wall is built using MetcoClad 718 filler material with the same strategy and conditions used in the previous tests. Afterwards, the wavy surface resulting from the LMD is processed via LBM up to a 0.5 mm depth. Finally, the desired regions are laser polished.

## 4. Conclusions

In the present work, a full laser-based manufacturing technique is proposed. According to the attained results, the following conclusions are drawn:

(1) The LBM process is proved capable of eliminating the waviness generated in the LMD process and enables to obtain a flat surface.
(2) Surface quality resulting from LBM may not comply with the desired requirements. However, high surface quality (N5–N6 roughness grade) is obtained after the LP stage.
(3) LP generates a recast layer with a thickness of 22 µm. Depending on the final application of the part, this circumstance must be considered, because it might be detrimental to the mechanical properties. Further investigations must be performed to determine the influence of this recast layer.
(4) In LBM a maximum material removal rate of 8.3 × 10^−3^ g·min^−1^ is obtained. Therefore, LBM is proved to be slow when compared with the machining processes. Consequently, the combination of LMD + LBM is only advantageous when difficult-to-cut materials are processed, or high-resolution detail operations are required.
(5) The LBM process is capable of manufacturing small details that may not be possible to attain with other traditional machining processes, such as milling.

## Figures and Tables

**Table 1:** Chemical composition (wt. %) of the MetcoClad 718 [24].

Cr	Mo	Nb	Fe	Ti	Si	Mn	C	B	Ni
19	3	5	18	1	0.2	0.08	0.05	0.005	Bal.

**Table 2:** LMD process parameters for the MetcoClad 718 [21].

Process Parameter	Value
Continuous wave laser power (W)	570
Scan velocity (mm·min^−1^)	525
Track offset (mm)	1.036
Overlap between tracks (%)	26
Powder mass flow (g·min^−1^)	8.78
Powder preheating temperature (°C)	60
Protective gas flow rate (L·min^−1^)	14

**Table 3:** Depth reached, in microns, after a single repetition for the different LBM process parameters.

Test Code	A	B	C	D	E
**0**	52.1	10.6	4.0	5.5	6.6
**1**	57.4	24.9	8.2	7.2	7.9
**2**	53.5	16.9	8.3	6.5	10.9
**3**	54.2	27.1	7.5	7.4	9.2
**4**	59.3	31.7	8.8	8.1	8.7
**5**	57.3	24.5	8.2	10.5	12.5

**Table 4:** LBM parameters for the MetcoClad 718.

Process Parameter	LBM
Mean pulse power (W)	6720
Velocity (mm·s^−1^)	800
Pulse frequency (Hz)	372,000
Pulse duration (ns)	20
Defocusing (mm)	0

**Table 5:** Hatching parameter values for LBM.

Process Parameter	LBM
Line spacing (mm)	0.05
Number of hatchings (-)	20
Angle increment (°)	17

**Table 6:** Ra value, in microns, after the different LP tests.

Test Code	0	1	2	3	4
**a**	1.12	0.98	0.79	0.87	1.54
**b**	0.95	0.90	0.67	0.77	1.42
**c**	0.91	0.89	0.68	0.75	1.38
**d**	1.15	1.03	0.83	0.72	1.61
**e**	0.98	0.93	0.72	0.72	1.45
**f**	0.93	0.92	0.75	0.62	1.41
**g**	1.34	1.18	0.95	1.04	1.89
**h**	1.14	1.04	0.80	0.89	1.70
**i**	1.09	1.05	0.82	0.90	1.74
**j**	1.36	1.24	0.95	0.86	2.03
**k**	1.18	1.10	0.86	0.85	1.74
**l**	1.10	1.10	0.90	0.74	1.78

**Table 7:** LP parameters for the MetcoClad 718.

Process Parameter	LP
Mean pulse power (W)	621
Velocity (mm·s^−1^)	100
Pulse frequency (Hz)	175,000
Pulse duration (ns)	460
Defocusing (mm)	4

**Table 8:** Hatching parameter values for LP.

Process Parameter	LP
Line spacing (mm)	0.02
Number of hatchings (-)	10
Angle increment (°)	36

**Table 9:** Arithmetic Mean Deviation of the Roughness Profile (Ra) in microns, according to ISO 4287. 0.25 mm Gaussian filter applied.

Measurement	LMD	LBM	LM
1	1.56	20.45	0.53
2	2.38	20.24	0.66
3	1.32	20.21	0.57
4	1.85	24.80	0.71
5	2.01	16.17	0.56
Average Ra	1.82	20.37	0.61

## Appendix A

Process parameters for LBM tests carried out to determine the optimal parameters are detailed in Table A1. In all tests, the hatching parameters are kept constant according to the values detailed in Table 4 (line spacing of 0.05 mm and 20 hatching with an angle increment of 17°). Test codes for the LBM tests are named with upper case letters.

**Table A1:** LBM process parameters.

Test	Velocity [mm·s^−1^]	Defocusing (mm)	Pulse Frequency [Hz]	Pulse Duration [ns]
A0	800	0	144,000	55
A1	800	0	201,000	37
A2	800	0	258,000	27
A3	800	0	315,000	23
A4	800	0	372,000	20
A5	800	0	429,000	17
B0	1100	0	144,000	55
B1	1100	0	201,000	37
B2	1100	0	258,000	27
B3	1100	0	315,000	23
B4	1100	0	372,000	20
B5	1100	0	429,000	17
C0	1400	0	144,000	55
C1	1400	0	201,000	37
C2	1400	0	258,000	27
C3	1400	0	315,000	23
C4	1400	0	372,000	20
C5	1400	0	429,000	17
D0	1700	0	144,000	55
D1	1700	0	201,000	37
D2	1700	0	258,000	27
D3	1700	0	315,000	23
D4	1700	0	372,000	20
D5	1700	0	429,000	17
E0	2000	0	144,000	55
E1	2000	0	201,000	37
E2	2000	0	258,000	27
E3	2000	0	315,000	23
E4	2000	0	372,000	20
E5	2000	0	429,000	17

## Appendix B

Process parameters for LP are shown in the following Table A2. In all tests, the line spacing is kept constant with a value of 0.02 mm. The angle increment between the hatchings is defined to sweep a total angle of 360° with the defined number of hatches. Test codes for the LP tests are named with lower case letters.

**Table A2:** LP process parameters.

Test	Velocity [mm·s^−1^]	Defocusing [mm]	Number of Hatches	Angle Increment between Hatchings [°]	Pulse Frequency [Hz]
a0	100	4	5	72	125,000
a1	100	4	5	72	150,000
a2	100	4	5	72	175,000
a3	100	4	5	72	200,000
a4	100	4	5	72	225,000
b0	100	4	10	36	125,000
b1	100	4	10	36	150,000
b2	100	4	10	36	175,000
b3	100	4	10	36	200,000
b4	100	4	10	36	225,000
c0	100	4	20	18	125,000
c1	100	4	20	18	150,000
c2	100	4	20	18	175,000
c3	100	4	20	18	200,000
c4	100	4	20	18	225,000
d0	100	5	5	72	125,000
d1	100	5	5	72	150,000
d2	100	5	5	72	175,000
d3	100	5	5	72	200,000
d4	100	5	5	72	225,000
e0	100	5	10	36	125,000
e1	100	5	10	36	150,000
e2	100	5	10	36	175,000
e3	100	5	10	36	200,000
e4	100	5	10	36	225,000
f0	100	5	20	18	125,000
f1	100	5	20	18	150,000
f2	100	5	20	18	175,000
f3	100	5	20	18	200,000
f4	100	5	20	18	225,000
g0	200	4	5	72	125,000
g1	200	4	5	72	150,000
g2	200	4	5	72	175,000
g3	200	4	5	72	200,000
g4	200	4	5	72	225,000
h0	200	4	10	36	125,000
h1	200	4	10	36	150,000
h2	200	4	10	36	175,000
h3	200	4	10	36	200,000
h4	200	4	10	36	225,000
i0	200	4	20	18	125,000
i1	200	4	20	18	150,000
i2	200	4	20	18	175,000
i3	200	4	20	18	200,000
i4	200	4	20	18	225,000
j0	200	5	5	72	125,000
j1	200	5	5	72	150,000
j2	200	5	5	72	175,000
j3	200	5	5	72	200,000
j4	200	5	5	72	225,000
k0	200	5	10	36	125,000
k1	200	5	10	36	150,000
k2	200	5	10	36	175,000
l3	200	5	10	36	200,000
l4	200	5	10	36	225,000
l0	200	5	20	18	125,000
l1	200	5	20	18	150,000
l2	200	5	20	18	175,000
l3	200	5	20	18	200,000
l4	200	5	20	18	225,000
